# Friends and Symptom Dimensions in Patients with Psychosis: A Pooled Analysis

**DOI:** 10.1371/journal.pone.0050119

**Published:** 2012-11-21

**Authors:** Domenico Giacco, Rose McCabe, Thomas Kallert, Lars Hansson, Andrea Fiorillo, Stefan Priebe

**Affiliations:** 1 Unit for Social and Community Psychiatry, Barts and The London School of Medicine and Dentistry, Queen Mary University of London, London, United Kingdom; 2 Department of Psychiatry, University of Naples SUN, Naples, Italy; 3 Klinik für Psychiatrie, Psychosomatik und Psychotherapie, Leipzig, Germany; 4 Department of Psychiatry and Psychotherapy, Dresden University of Technology, Dresden, Germany; 5 Department of Clinical Neuroscience, Lund University, Lund, Sweden; Baylor College of Medicine, United States of America

## Abstract

**Background:**

Having friends is associated with more favourable clinical outcomes and a higher quality of life in mental disorders. Patients with schizophrenia have fewer friends than other mentally ill patients. No large scale studies have evaluated so far what symptom dimensions of schizophrenia are associated with the lack of friendships.

**Methods:**

Data from four multi-centre studies on outpatients with schizophrenia and related disorders (ICD F20-29) were included in a pooled analysis (N = 1396). We established whether patients had close friends and contact with friends by using the equivalent items on friendships of the Manchester Short Assessment of Quality of Life or of the Lancashire Quality of Life Profile. Symptoms were measured by the Brief Psychiatric Rating Scale or by the identical items included in the Positive and Negative Syndrome Scale.

**Results:**

Seven hundred and sixty-nine patients (55.1%) had seen a friend in the previous week and 917 (65.7%) had someone they regarded as a close friend. Low levels of negative symptoms and hostility were significantly associated with having a close friend and contact with a friend. Overall, almost twice as many patients with absent or mild negative symptoms had met a friend in the last week, compared with those with moderate negative symptoms.

**Conclusions:**

Higher levels of negative symptoms and hostility are specifically associated with the lack of friendships in patients with psychotic disorders. These findings suggest the importance of developing effective treatments for negative symptoms and hostility in order to improve the probability of patients with schizophrenia to have friends.

## Introduction

Friendship can be defined as a “distinctively personal relationship that is grounded in a concern on the part of each friend for the welfare of the other, for the other’s sake, and that involves some degree of intimacy” [1]. The definition of friendship can vary significantly in different geographical and cultural contexts but also due to personal factors, (attachment style, gender, previous experiences) [2]. Yet, the role of friends as a source of social support is becoming increasingly important in contemporary society [3]–[4], as a consequence of changes in family structure and of the increased number of people living alone [5]. It has been widely recognized that having friends provides patients with a mental disorder with emotional and practical support and helps them to cope with life stressors [6]. Relationships with friends may also positively affect physical and mental health by improving health behaviours and help seeking and confer psychological benefits for depression, self efficacy, self esteem, coping, and morale [7].

People with psychotic disorders tend to have fewer friends and social relationships compared to the general population and to patients with other mental and physical disorders [2]–[8].

While many factors, such as deficits in neurocognition and social cognition, unemployment, financial difficulties and stigma are likely to reduce patients’ social functioning [9]–[11], different symptoms of psychotic disorders have been linked to patients’ difficulties in establishing and maintaining social contacts. Social withdrawal of patients with psychotic disorders has been suggested to be an attempt at avoiding excessive stimulation and subsequently relapse. Hansen and colleagues [12] proposed a distinction between passive social withdrawal, which may be mostly related to negative symptoms, and active social avoidance, which has been linked to positive symptoms. The lack of motivation which is part of the negative symptoms dimension may play a significant role in reducing contact with friends [13]. As regards the other symptom domains of psychosis, depressive and anxiety symptoms may reduce patients’ drive towards social activities and contacts [14]–[15]; thought disorders may influence patients’ language and ability to share their thoughts and feelings with others [6]; high levels of excitement and activation may make patients appear unpredictable and dangerous so that others avoid contact and longer relationships with them [16]; high levels of hostility have been found to predict worse social integration, defined as number of contacts and significant relationships [17].

However, although many studies have assessed the relationship between psychotic symptoms and patients’ global social networks, few data are available on the associations of symptoms specifically with friendship, with its characteristics of an intimate and supportive relationship.

To our knowledge, only one mixed-methods study, carried out on 151 patients with schizophrenia in south England, has specifically focused on relationships with friends of patients with schizophrenia [2] finding an association between levels of both positive and negative symptoms and contacts with a friend. Other studies [18]–[19] found a moderate correlation of negative symptoms and hostility with social functioning and involvement in leisure activities in the community that were correlated with friendships in relatively small samples (n = 56 and n = 263, respectively).

Evidence from larger samples is necessary to further understand how different symptoms are specifically associated with contacts with friends. Given the protective effects of friendship, interventions to improve patients’ friendships and, as a consequence, social support, clinical outcomes, and quality of life may need to consider specific symptom dimensions.

This study assessed, through a pooled analysis of individual patient data from four Europe-wide multicentre studies, the association of five symptom dimensions of psychotic disorders (negative symptoms, thought disorders, depression/anxiety symptoms, activation and hostility) with having a close friend and contacts with friends in the community. As age and gender have been found to be associated with patients’ social contacts in previous studies [20]–[22], we also investigated whether the associations between symptoms and having a friend and contacts with friends were similar for males and females and in different age groups. Initially, our analysis included all patients with psychotic disorders, diagnosed according to ICD-10 criteria (F20–29). In a second phase, we performed a sensitivity analysis including only patients with schizophrenia (F20).

## Methods

### Sample

For this study we analysed data from four multi-centre studies, i.e. one cluster randomized controlled trial and three prospective observational studies.

The DIALOG study [23] was a cluster randomized controlled trial testing the effect of a computer mediated intervention structuring patient-clinician communication in community mental health care. It was conducted with outpatients at sites in six European countries (United Kingdom, Spain, Netherlands, Sweden, Germany, Switzerland). The baseline data were analysed (n = 502), which were obtained before randomization and hence not affected by the study intervention.

The “Nordic multicentre study” [24] was a multi-centre cross-sectional study of subjective quality of life in people with schizophrenia living in the community, carried out in five Scandinavian countries (Sweden, Denmark, Finland, Iceland, Norway). Data from 341 patients were included in the analysis.

The “EUNOMIA” study [25] was an observational prospective study in 12 European countries to assess outcomes of coercive treatments and influencing factors. In our study, we took data from the last follow-up interview (three months after admission) to have a large sample of patients who had been discharged and were living in the community. Only data from centres where evaluation of patients’ quality of life had been performed through Manchester Short Assessment of Quality of Life (MANSA) could be included in the analysis (Germany, Czech Republic, Lithuania, Poland, Slovakia, Sweden) (n = 352). UK data were excluded to avoid duplicates with the InvolvE [26] study that was conducted in the same period in the same inpatient wards.

The “InvolvE” study [26] was an observational prospective study on outcomes of involuntary hospitalizations in 22 hospitals in England. For our study we took data from the last follow-up assessment (1 year after the index admission), again to have a large sample of patients who lived in the community (n = 201).

Rationale, methods and findings of the studies have been published elsewhere [23]–[26]. The inclusion criteria for patients assessed in the course of these four studies were: 1) having a diagnosis of schizophrenia, schizotypal, or delusional disorders (F20–F29) according to ICD-10 [27]; diagnoses were made using ICD-10 criteria because they are broader than DSM-IV criteria and include all range of psychotic disorders (i.e. schyzotypal disorder is included among psychotic disorders in ICD-10 whilst in DSM-IV it is considered a personality disorder) [28]; 2) not having been hospitalized in the seven days before the interview, in order to assess friendships in the community using questions relating to behaviour in the last week; 3) having responded to the relevant items on the Manchester Short Assessment of Quality of Life (MANSA) and the Lancashire Quality of Life Profile (LQOLP).

### Ethics Statement

All studies included in this pooled analysis have obtained approval of relevant ethics committees, and all patients provided written informed consent.

### Measures

Studies used either the Lancashire Quality of Life Profile (LQOLP) or the Manchester Short Assessment of Quality of Life (MANSA) instruments which contain equivalent items for assessing patients’ friendships [29]–[30]. For the purposes of our study, we analysed two items on friendships from MANSA and LQOLP. One reflected a behavioural criterion (“have you seen a friend within the last week, i.e. visited a friend, been visited by a friend, or met a friend outside both your home and work?”), and the other a subjective appraisal of friendship (“do you have anyone whom you would call a close friend?”). We chose to study both a subjective and an objective criterion of friendship because we wanted to assess contacts with friends from both perspectives. Some patients may report having close friends, but not feel able to actually see them due to symptoms like motor retardation, lack of motivation or depressive symptoms. Other patients with specific symptoms (i.e. paranoid delusions or suspiciousness) may report no close friends even though their social network is not totally compromised. Both questions are answered with no ( = 0) or yes ( = 1). We focused on these two questions as they are brief, straightforward to understand, and simple to answer so that a wide range of patients can respond including those with high symptom levels and low motivation. This was seen as important to limit a selection bias with lower response rates in highly symptomatic patients. Data on these questions were available in all data sets allowing us to take full advantage of a pooled analysis.

In two studies [25]–[26] symptoms were assessed on the 24-item Brief Psychiatric Rating Scale [31] and in the other two [23]–[24] on the Positive and Negative Syndrome Scale (PANSS) [32]. PANSS items used in this analysis are identical to BPRS items. The Cohen kappa’s values for the inter-rater reliability of symptom assessments on these scales ranged from 0.71 to 0.90 in the four studies. This allowed computation of BPRS-18 items and scores of five BPRS-18 subscales: 1) anxiety/depression (items: anxiety, guilt, depression, somatic); 2) negative symptoms (items: blunted affect, emotional withdrawal, motor retardation); 3) thought disorders (items: thought content, conceptual disorganization, hallucinatory behavior, grandiosity); 4) activation (items: excitement, tension, mannerisms-posturing); and 5) hostility (items: hostility, uncooperativeness, suspiciousness) [33]. Additionally we obtained data on patients’ age and gender from all studies.

### Statistical Analysis

Stata 12 for Windows was used for all data analyses [34]. Descriptive statistics for the distribution of all considered variables in the total sample were calculated.

Four datasets were pooled to identify main effects of symptom domains and also interaction effects of symptom domains with age, gender or both. A “pooled analysis” [35]–[36] was conducted. This approach enables a more precise estimate of effects of influential factors and takes into account confounding factors such as patients’ age and gender and the heterogeneity of centres. A further advantage is that the same statistical model could be used with data from methodologically diverse studies and to test interactions with specific patients’ characteristics (in this case age and gender).

The correlation between the behavioural item, i.e. contact with friends in the last week, and the subjective item, i.e. patients’ subjective appraisal of having a close friend was explored by the phi test. Univariable and multivariable logistic mixed models, adjusted for heterogeneity across centres and studies, were used to identify the factors associated with behavioural and subjective item for friendship. The multivariable model, adjusted for confounding factors (patients’ age and gender), included all BPRS subscales. In this three-level model, patient-level measurements (level-1) were treated as nested within centres (level-2), and centres as nested within studies (level-3). To illustrate the size and clinical relevance of possible associations between symptom domains and friendship, we divided average scores of the given BPRS subscale in six intervals (i.e. 1, from 1 to 2, 2 to 3, 3 to 4, 4 to 5, 5 to 6) and showed the percentages of patients who had seen a friend in the last week and had a close friend for each symptom interval. For each interval, the higher number was included in the lower interval (i.e. 2 was included in the interval from 1 to 2) and there were no values higher than 6. Then, dichotomous variables were created for each subscale that had a statistically significant association with the two friendship items. In these dichotomous variables all the values of BPRS subscales that were lower than 2 (with 2 included) were coded as “1″ and all the values that were higher than 2 as “0″. The univariable associations of these variables with friendship items were tested by mixed logistic regression models, adjusted for heterogeneity of centres and studies.

Two-way interactions for age and gender were tested to establish whether they influenced associations between BPRS subscales and patients’ contact with friends. Statistical significance of interaction terms was assessed using Wald tests.

Since the sample contained patients with different diagnoses within the spectrum of schizophrenia and related disorders, we conducted a sensitivity analysis, repeating the analyses in the pooled sample only with those patients who had a diagnosis of schizophrenia (F20 according to the ICD-10).

## Results

### Patient Characteristics

Across the studies, a total of 1396 patients met the inclusion criteria (n = 502 from the DIALOG study; n = 341 from the Nordic Multicentre study; n = 352 from the EUNOMIA study; n = 201 from the InvolvE study). Patients were predominantly male (844, 60.5%), with a mean age of 39.9 years (SD = 11.1). The age span in years was 18–64 in EUNOMIA study (median = 39, quartiles = 29–49), 18–65 in DIALOG study (median = 42, quartiles = 33.5–50), 18–64 in the INVolvE study (median = 36, quartiles = 26–45) and 20–55 in the Nordic Multicentre study (median = 40, quartiles = 32–46).

Seven hundred and sixty-nine patients (55.1%) had seen a friend in the previous week and 917 (65.7%) had someone they regarded as a close friend. Overall patients showed low scores on different BPRS subscales and the distribution of these scores were skewed to the left. The mean scores of the BPRS subscales were: 2.1 (SD = 0.9) on the depression/anxiety subscale; 1.9 (SD = 0.9) on the negative symptoms subscale; 1.9 (SD = 1.0) on the thought disorders subscale; 1.5 (SD = 0.6) on the activation subscale; 1.5 (SD = 0.7) on the hostility subscale.

The main socio-demographic and clinical characteristics of the individual studies and samples and of the pooled sample are reported in Table 1.

**Table 1:** Characteristics of the four included studies and samples.

Study sample	DIALOG study^19^	Nordic multicentre study^20^	EUNOMIA study^21^	InvolvE study^22^	Total sample
**Study sites**	UK, Spain, Netherlands,Sweden, Germany,Switzerland	Sweden, Denmark,Finland, Iceland,Norway	Germany, Poland, Slovakia,Czech Republic, Lithuania,Sweden	England	-
**Sample size** 1	502	341	352	201	1396
**Measure** 2	MANSA	LQOLP	MANSA	MANSA	–
**Study design**	Randomized controlled trial	Prospective-observational	Prospective-observational	Prospective-observational	–
**Friend seen in the** **last week, yes, n (%)**	303 (60.4)	159 (46.5)	205 (58.2)	103 (51.2)	769 (55.1)
**Close friendship available, yes, n (%)**	321 (63.9)	223 (65.2)	239 (67.9)	135 (67.2)	917 (65.7)
**Patients’ age, mean (sd)**	42.1 (11.4)	38.9 (8.7)	39.7 (11.8)	36.5 (11.4)	39.9 (11.1)
**Patients’ gender, female, n (%)**	169 (33.7)	133 (38.9)	192 (54.5)	59 (29.4)	552 (39.5)
**BPRS – anxiety/depression subscale score, mean (sd)**	2.3 (0.9)	2.3 (0.9)	1.8 (0.8)	2.1 (1.0)	2.1 (0.9)
**BPRS – negative symptoms subscale score, mean (sd)**	2.1 (0.9)	2.0 (0.8)	1.7 (0.8)	1.7 (0.8)	1.9 (0.9)
**BPRS – thought disorders subscale score, mean (sd)**	2.1 (1.0)	2.0 (1.0)	1.4 (0.6)	1.8 (1.1)	1.9 (1.0)
**BPRS – activation subscale score, mean (sd)**	1.4 (0.6)	1.9 (0.8)	1.3 (0.4)	1.4 (0.6)	1.5 (0.6)
**BPRS – hostility subscale score, mean (sd)**	1.4 (0.6)	1.7 (0.8)	1.4 (0.6)	1.7 (0.9)	1.5 (0.7)

1 Sample size refers to included patients with an ICD-10 clinical diagnosis of schizophrenia, schizotypal, or delusional disorders, for which BPRS-18 and MANSA/LQOLP items on friendship scores were available.

2 LQOLP, Lancashire Quality of Life Profile; MANSA, Manchester Short Assessment of Quality of Life.

### Associations of Symptom Domains and Friendship Items

The behavioural (having seen a friend) and subjective (having a close friend) items on friendship were significantly correlated (phi = .589, p<.001).

#### a. Contact with friends in the previous week

As shown in Table 2, the scores of all BPRS subscales were univariably associated with fewer contacts with friends in the last week. However, when controlling for all BPRS subscales, patients’ age and gender and adjusting for random effects of heterogeneity of centres and studies, only higher levels of negative symptoms and hostility were associated with patients’ contact with friends in the previous week with odds ratios (OR) of.693 (95% confidence interval (95% CI) = .602–.797, p<.001) and.823 (95% CI = .680–.996, p = .046), respectively; younger patients more often had contacts with friends in the previous week (OR age = 0.980, 95% CI = 0.970–0.990, p<.001). The multivariable model is reported in Table 3.

**Table 2:** Univariable mixed model analyses of associations between symptoms and having seen a friend in the last week as a dependent variable adjusted for studies and centres within studies (21 centres, 4 studies, 1396 patients).

	Odds ratio	Odds ratio(95% CI^a^)	P
BPRS - depression/anxiety subscale	.856	.759–.966	.012
BPRS - negative symptoms sub scale	.618	.538–.710	<.001
BPRS - thought disorder sub scale	.806	.715–.907	<.001
BPRS - activation sub scale	.690	.570–.835	<.001
BPRS - hostility sub scale	.714	.607–.840	<.001
Patients’ age	.979	.970–.989	<.001
Patients’ gender	.810	.648–1.013	.065

a CI = Confidence Interval.

**Table 3:** Multivariable mixed model analysis of associations between symptoms and having seen a friend in the last week as a dependent variable adjusted for studies and centres within studies (21 centres, 4 studies, 1396 patients).

	Adjusted for BPRS-18 subscales, patients’ age and gender		
	Odds ratio	Odds ratio (95% CI^a^)	P
BPRS - depression/anxiety subscale	.983	.860–1.124	.804
BPRS - negative symptoms sub scale	.693	.602–.797	<.001
BPRS - thought disorder subscale	.971	.847–1.114	.679
BPRS - activation sub scale	.870	.708–.1.070	.186
BPRS - hostility sub scale	.823	.680–.996	.046
Patients’ age	.980	.970–.990	<.001
Patients’ gender	.835	.659–1.058	.135
Sigma_u	.226	.093–.551	
Rho	.015	.003–.084	

a CI = Confidence Interval.

#### b. Having a close friend

The univariable and multivariable models that tested the associations between symptom domains and the subjective appraisals of patients of having a close friend are reported in Table 4 and Table 5.

**Table 4:** Univariable mixed model analyses of association between symptoms and likelihood of having a close friend as a dependent variable adjusted for studies and centres within studies (21 centres, 4 studies, 1396 patients)^a^.

	Odds ratio	Odds ratio(95% CI^a^)	P
BPRS - depression/anxiety subscale	.902	.795–1.023	.108
BPRS - negative symptomssub scale	.638	.555–.734	<.001
BPRS - thought disorder sub scale	.823	.729–.930	.002
BPRS - activation sub scale	.761	.629–.921	.005
BPRS - hostility sub scale	.712	.604–.838	<.001
Patients’ age	.984	.974–.994	.002
Patients’ gender	.732	.577–.930	.011

a CI = Confidence Interval.

**Table 5:** Multivariable mixed model analysis of association between symptoms and likelihood of having a close friend as a dependent variable adjusted for studies and centres within studies (21 centres, 4 studies, 1396 patients).

	Adjusted for BPRS-18 subscales, patients’ age and gender		
	Odds ratio	Odds ratio (95% CI^a^)	P
BPRS - depression/anxiety subscale	1.005	.872–1.158	.944
BPRS - negative symptoms sub scale	.676	.583–.783	<.001
BPRS - thought disorder subscale	.960	.832–1.107	.574
BPRS - activation sub scale	.963	.772–.1.200	.738
BPRS - hostility sub scale	.813	.670–.988	.037
Patients’ age	.983	.972–.994	.002
Patients’ gender	.758	.588–.977	.032
Sigma_u	.290	.153–.547	
Rho	.025	.007–.083	

a CI = Confidence Interval.

Higher levels of all the symptom domains, with the exception of depression/anxiety symptoms, were univariably associated with the absence of a close friend. When controlling for age and gender and adjusting for heterogeneity of centres and studies, only the associations of the absence of close friendships with higher levels of negative symptoms (OR = .676; 95% CI = .583–.783; p<.001) and hostility (OR = .813; 95% CI = .670–.988; p = .037) held true. Younger patients were more likely to have a close friend (OR age = .983; 95% CI = .972–.994; p = .002). Male patients reported less frequently than female patients that they had a close friendship (OR = .758; 95% CI = .588–.977; p = .032).

### Friendship Items and BPRS Sub-scales Intervals

The number and percentage of patients who had contact with friends in the previous week and who stated that they had a close friend are reported in Table 6 and Table 7 for each of the six intervals of the BPRS negative symptoms and BPRS hostility subscales.

**Table 6:** Contacts with friends of patients with different scores on the BPRS negative symptoms and hostility subscales.

BPRS subscales score	Have you seen a friend in the last week?					
	Negative symptoms			Hostility symptoms		
Intervals	Total^a^ (n)	Yes^b^ (n)	Yes^c^ (%)	Total^a^ (n)	Yes^b^ (n)	Yes^c^ (%)
1	321	214	66.7	550	328	59.6
>1 and < = 2	545	311	57.1	610	336	55.1
>2 and < = 3	369	177	48.0	179	84	46.9
>3 and < = 4	125	48	38.4	41	14	34.1
>4 and < = 6	19	7	36.8	9	2	22.2

a Number of patients at each interval of BPRS subscales score.

b Number of patients at each interval of BPRS subscales score who reported to have seen a friend in the last week.

c Percentage of patients at each interval of BPRS subscales score who reported to have seen a friend in the last week.

**Table 7:** Percentage of patients with different scores on the BPRS negative symptoms and hostility subscales who have a close friend.

BPRS subscales score	Do you have anyone you would call a close friend?					
	Negative symptoms			Hostility symptoms		
Intervals	Total^a^ (n)	Yes^b^ (n)	Yes^c^ (%)	Total^a^ (n)	Yes^b^ (n)	Yes^c^ (%)
1	321	247	77.7	550	392	71.3
>1 and < = 2	545	366	67.2	610	388	63.6
>2 and < = 3	369	216	58.5	179	105	58.7
>3 and < = 4	125	67	53.6	41	21	55.3
>4 and < = 6	19	8	42.1	9	5	55.6

a Number of patients at each interval of BPRS subscales score.

b Number of patients at each interval of BPRS subscales score who reported to have a close friend.

c Percentage of patients at each interval of BPRS subscales score who reported to have a close friend.

#### a. Negative symptoms

Patients with very low levels of negative symptoms (lower than “2” at BPRS negative symptoms subscale) had almost double the odds of having met a friend in the previous week (OR = 1.745; IC 95% = 1.399–2.176, p<.001) and of having a close friend (OR = 1.838; IC 95% = 1.461–2.313, p<.001) compared to those with higher levels of negative symptoms. The odds ratios are adjusted for heterogeneity of centres and studies.

#### b. Hostility

Patients with very low levels of hostility (lower than “2” on BPRS hostility symptoms subscale) had higher odds of having met a friend in the previous week (OR = 1.520; 95% CI = 1.139–2.028, p = .004) and of having a close friend (OR = 1.498; 95% CI = 1.117–2.011, p = .007) compared to those with higher levels of hostility. The odds ratios are adjusted for heterogeneity of centres and studies.

#### c. Patients with low or moderate-high levels of both negative symptoms and hostility

Among the 770 patients who had very low or absent levels both of negative symptoms and hostility (BPRS subscale scores lower than two), 477 (61.9%) had seen a friend in the last week and 549 (71.3%) had someone they regarded as a close friend.

Patients with at least low-moderate levels of both negative symptoms and hostility (BPRS subscales score higher than two) were 138. Among them, 57 (40.6%) had seen a friend in the last week and 73 (52.1%) had someone they regarded as a close friend.

### Interaction Effects of Symptoms with Age and Gender on the Associations with Friendship Items

#### Contacts with friends in the previous week

The association of higher levels of negative symptoms with contacts with friends in the last week was neither influenced by age (**Wald test** value Z = −0.35, p = .724) nor by gender (Z = −1.49, p = .135).

Similarly, no influence of age and gender on the association of higher levels of hostility with contacts with friends was found (Wald test values were Z = 0.75, p = .452 for age and Z = 1.73, p = .084 for gender, respectively).

### Having a Close Friend

The association of higher levels of negative symptoms with having no close friends was not influenced by age (Z = −0.60; p = .546) or gender (Z = −0.25; p = .806). The interaction of gender on the association of hostility with having no close friends was not statistically significant (Z = 1.28; p = .201). However, in younger patients the association between hostility and absence of close friendships was stronger (Z = 2.68, p = .007).

### Sensitivity Analysis only with Patients with Schizophrenia

When we repeated the analyses in the pooled sample only with those patients who met the criteria for schizophrenia (F20) (n = 1019), the association of higher levels of negative symptoms and hostility with contacts with friends in the last week and the subjective appraisals of having a close friend remained statistically significant. No other symptom domain was significantly associated with friendships in the multivariable analysis. No interactions between symptoms and socio-demographic variables were found. In particular, the interaction between hostility levels and age, that was statistically significant in the global sample, failed to reach statistical significance in the subsample of patients with schizophrenia.

## Discussion

### Main Results

This is the largest study to date analyzing how specific psychotic symptoms are associated with social contacts of patients with schizophrenia related disorders and the first one focussing specifically on friendships, as a specific and relevant sub-category of social contacts.

Higher levels of negative symptoms and hostility are associated with fewer contacts with friends and absence of close friendships. The association between negative symptoms and contacts with friends is more marked in male patients. Depression/anxiety symptoms, thought disorders and levels of activation were only univariably associated with patients’ friendships. When the associations were adjusted for the influence of other symptoms, no significant association was found between depression/anxiety symptoms, thought disorders and activation and friendships.

Despite the suggestion that all symptoms of schizophrenia are likely to have an impact on patients’ social relationships [6], [9], [12]–[19], this study showed that levels of negative symptoms and hostility are specifically associated with the disruption of more intimate social relationships, i.e., friendships, that can be important sources of social support. The univariable association of other symptoms with friendship may mainly reflect the overall severity of psychotic symptoms.

### Comparison with the Available Literature

We found a high number of patients did not have a close friend and did not see any friend in the previous week, which is in line with the previous literature [2]–[8]. The proportion of patients with psychosis without close friends is much higher than in the general population (25% in a representative survey carried out in the United States [37]). Our results suggest that the lack of friendships in these patients is more related to negative symptoms and hostility than to depressed mood, “active avoidance” and thought/perception disorders.

The role of hostility in influencing the size of patients’ social networks and their social functioning has already been reported by other studies [17]. Our findings indicate that hostility levels may also have an impact on closer relationships such as friendships. As shown by the statistically significant interactions between age and hostility levels in the global sample (not in the subsample of patients with F20 diagnosis), such impact might be particularly strong in the early phase of the disorders and in younger patients.

While the association of hostility levels with contacts with friends may look rather intuitive, the link of negative symptoms with friendships probably deserves some further exploration. Cognitive-behavioural models [38] have suggested that social withdrawal may constitute a “coping strategy” for reducing stress and arousal levels. Such coping strategy, characterized by symptoms such as blunted affect and emotional withdrawal, may limit current distress; however, it could be maladaptive in the long term leading to increased social isolation and poorer clinical and social outcomes [2]. Furthermore, other symptoms included in the negative symptom dimension, such as lack of motivation and motor retardation, may reduce social engagement of these patients and hamper their contacts even with people they consider as close friends [13].

High levels of activation (i.e. excitement, tension, mannerism and posturing) and thought disorders (i.e. thought content disorders, conceptual disorganization, hallucinations and grandiosity) might lead other people to believe that patients are unpredictable and, possibly, dangerous [16]. The results of this study, however, do not support the hypothesis that levels of activation and thought disorders determine patients’ difficulties in having friends. In contrast to previous studies which established a link between depressive symptoms in schizophrenia and deficits in object relations and reality testing [39], a high level of anxiety/depression symptom dimension was not associated with less contacts with friends when controlling for other symptom dimensions effect.

In our sample, younger patients were more likely to have had recent contacts with friends than older patients. This finding might be interpreted as a consequence of the progressive deterioration of one’s social network related to psychotic disorders [40] that may finally also affect the more intimate relationships, such as friendship.

Male patients had less frequently someone they regarded as a close friend. This may be due to a greater fear of intimacy and lower levels of emotional commitment in relationships of males, documented in studies on clinical and non-clinical populations [21], [41] and related to culturally determined gender attitudes [42]. Substance misuse may also play a role in reducing social contacts amongst male patients with psychosis, due to its higher prevalence amongst this patient group and its association with higher symptom levels [43].

### Implications

Although the cross-sectional nature of the study does not allow conclusions to be drawn on causal relationships, we found significant associations of higher levels of negative symptoms and hostility with lack of friendships. Despite suggestions that all symptoms of psychotic disorders can have a role in patients’ difficulties in establishing and maintaining social relationships, the levels of negative symptoms and hostility may be specifically associated with the disruption of more intimate social relationships, i.e. friendships, that can be important sources of social support. It might be hypothesized that a full reduction of moderate to severe symptom levels in these two domains might have a relevant impact on the patient’s chances to have and meet friends.

There is limited evidence on the effectiveness of available treatments specifically for hostility. A range of antipsychotics have been suggested as effective [44] in reducing hostility, but patients with high levels of hostility symptoms frequently show a reduced adherence to antipsychotic medication [45].

On the other hand, the treatment of negative symptoms is particularly challenging. Intensive psychosocial treatment has been found to have a beneficial effect on negative symptoms [46]. However, pharmacological and psychosocial therapies that are usually available in mental health services show limited effectiveness on this symptom dimension and the full remission is rarely achieved in practice [47]–[48].

The limits in effectiveness of available therapies may pose patients with high levels of these symptoms at high risk of poorer psychological and physical health outcomes [7].

Furthermore, even among patients with no or very low levels of both negative symptoms and hostility, about 38% did not see a friend in the last week and 29% reported not having a close friend. It is possible that other factors, such as impairment of neuro-cognitive performance and deficits in social cognition [49], may have a negative impact on social relationships of these patients. Neurocognitive deficits such as significant impairments in the domains of processing speed, verbal memory, executive function, working memory, sustained attention and language can be present in patients with psychotic disorders from the premorbid phase onwards [50]. They may have a negative impact on establishing social networks in the adolescence and early adulthood [51]. Also deficits in social cognition that are present in patients with psychosis [49] may have a negative influence on their social relationships independently from symptom severity. Finally, the secondary consequences of psychotic disorders such as unemployment, stigma and financial problems may reduce the opportunities for these patients to engage in social activities and establish relationships [11].

### Strengths and Limitations

The large sample size of this study provided a substantial statistical power for multivariable tests, including the testing of interaction effects, and ensured the validity and generalisability of both positive and negative findings. This is also the first multicentre assessment of friendship as an intimate relationship, with its potential to provide support to patients [6]. Both subjective and behavioural items related to friendship were included and their associations with symptoms were consistent. The assessments were conducted in international multi-centre studies carried out in different European countries on patients living in the community. The analysis considered the possible heterogeneity of findings between centres and studies. The inter-rater reliability of clinical symptom assessments was high in all the studies (Cohen Kappa’s values between 0.71 and 0.90).

However, some limitations should be noted: a) Study patients were not representative of all patients in the given service. Selection biases might have influenced the final scores of items on friendship and of BPRS subscales. Nevertheless, the aim of the study was to assess associations between clinical symptoms and patients’ contacts with friends, and associations are usually more robust towards selection bias than absolute levels [52]; b) The definition of friendship was not provided to patients “a priori” but was mainly based on patients’ own assessment of what friendship is, which may vary among different individuals. Characteristics of the nature of the friendship were not assessed, which might have led to an over-estimation of the behavioural criterion. We cannot exclude that friends seen in the last week might have been in some cases simple acquaintances. However the correlation between subjective and behavioural criteria and their association with the same symptom domains contributes to confirm the validity of these findings; c) Only patients with non-affective psychotic disorders were included in this analysis; d) All included studies were conducted in Europe, and the generalizability of the findings to samples outside Europe needs to be explored; e) the associations of friendship items with other variables such as stigma, neurocognitive and social cognition disorders and secondary effects of schizophrenia (unemployment, deficit in social motor coordination, financial problems, stigma) [9]–[12], [53] could not be explored; f) duration of illness and type of treatment received by patients were not assessed in all included studies, therefore we could not test the effects of these variables. However, patients’ age may be regarded as a proxy variable for the duration of illness; g) Patients were not in an acute phase and were living in the community when the assessments were performed. Consequently, the BPRS scores were generally low. We cannot exclude a possibility that the contacts with friends may change significantly during acute phases and fluctuate over the course of illness. It might even be the case that different symptom dimensions are associated with relationship with friends in the acute phases of psychotic disorders.

### Conclusions

Treatment of negative symptoms and hostility may be important for enabling patients with psychotic disorders to engage in friendships. However, the limited effectiveness of currently available treatments for negative symptoms, the reduced adherence to treatment of patients with high levels of hostility, and the significant number of patients that do not have friendships despite low levels of negative symptoms and hostility, suggest that further therapeutic interventions and support need to be developed to address the difficulties of patients with schizophrenia in establishing and maintaining friendships.

Experimental studies are required to longitudinally explore the correlations between symptom domains of psychotic disorders and friendships and to assess to what extent effective treatment of negative symptoms and hostility might indeed be followed by more patient friendships.

## Supporting Information

Checklist. S1 (DOC)
